# Preliminary psychometric validation of the Russian version of the vividness of tactile imagery questionnaire (VTIQ-RUS)

**DOI:** 10.3389/fpsyg.2026.1814960

**Published:** 2026-05-20

**Authors:** Lev Yakovlev, Evgeniya Rakova, Andrei Miroshnikov, Aigul Nasibullina, Nikolay Syrov

**Affiliations:** 1 Faculty of Biology, Shenzhen MSU-BIT University, Shenzhen, China; 2 Department of Human and Animal Physiology, Faculty of Biology, Lomonosov Moscow State University, Moscow, Russia; 3 Center for Bio- and Medical Technologies, Skolkovskij Institut Nauki i Tehnologij, Moscow, Russia; 4 Computational Neurology Group, Ruhr University Bochum, Bochum, Germany

**Keywords:** imagery vividness, mental imagery, psychometric validation, questionnaire, tactile imagery

## Abstract

There is a lack of validated standardized measures for tactile imagery vividness. Although the original Vividness of Tactile Imagery Questionnaire (VTIQ) has been introduced, validated adaptations for non-English-speaking populations remain limited. Therefore, the present study aimed to translate and adapt the VTIQ for Russian-speaking populations (VTIQ-RUS) and provide a preliminary psychometric evaluation. One hundred and fifty healthy adults completed the 16-item questionnaire twice with a two-week interval. Conducted analysis demonstrated high internal consistency (Cronbach’s *α* = 0.93) and strong test–retest reliability over the two-week interval (Spearman’s *ρ* = 0.791, *p* < 0.001). Inter-item correlations were moderate (range = 0.241–0.608), indicating coherent but non-redundant item relationships. Exploratory factor analysis supported a unidimensional structure, with all items loading moderately to strongly on a single factor (0.564–0.774). These findings provide preliminary evidence that the VTIQ-RUS is a reliable and structurally coherent instrument for assessing tactile imagery vividness. However, confirmatory factor analysis and construct validity were not assessed, and therefore further validation is required.

## Introduction

Mental imagery is the ability to generate and manipulate sensory experiences in the absence of external stimuli (Kosslyn et al., 2001; Pearson, 2019). The vividness of mental imagery varies across individuals and across sensory modalities, influencing both subjective experience and underlying neural processes. Assessing imagery vividness therefore provides a crucial bridge between phenomenology and objective measures, offering insights into how internal models of the world are constructed and maintained. In the visual domain, subjective ratings of imagery vividness have been shown to correlate with activity in the visual cortex, demonstrating a direct relationship between subjective experience and neural underpinnings (Amedi et al., 2005; Cui et al., 2007).

Tactile imagery represents a distinct modality of mental imagery, involving the internal simulation of somatosensory experiences such as pressure, texture, and temperature. Unlike visual imagery, which has been extensively studied and operationalized, tactile imagery remains comparatively underexplored, despite evidence suggesting its involvement in sensorimotor integration and somatosensory processing (Yao et al., 2022; Yakovlev et al., 2023, 2024, 2026). Importantly, individual differences in tactile imagery vividness may influence both perceptual processing and neural activation patterns, highlighting the need for reliable tools to quantify this construct.

Standardized self-report measures have been developed to assess individual differences in imagery vividness across other sensory modalities, including visual, auditory, olfactory, and motor imagery (Marks, 1973, 1995; Isaac et al., 1986; Gissurarson, 1992; Gilbert et al., 1998; Malouin et al., 2007). However, comparable validated tools for assessing tactile imagery at the individual level have been lacking, which has limited systematic investigation of its phenomenology and its relationship to behavioral and neural measures.

To address this gap, Nierhausva et al. (2023) proposed the Vividness of Tactile Imagery Questionnaire (VTIQ), a 16-item instrument using a 5-point Likert scale to assess the vividness of imagined tactile scenarios. However, the psychometric properties of the VTIQ have not yet been extensively evaluated across independent samples and cultural contexts. In particular, validated adaptations of the instrument for non-English-speaking populations are currently lacking, and no instrument for assessing tactile imagery vividness has been adapted for Russian-speaking populations.

Given the absence of validated instruments for assessing tactile imagery vividness in Russian-speaking populations, there is a clear need for cross-cultural adaptation and psychometric evaluation of the VTIQ. Establishing the reliability and measurement stability of a Russian version is a necessary first step before the instrument can be applied in experimental or neurophysiological research. Moreover, standardized assessment tools are essential for ensuring comparability across studies and for investigating relationships between subjective imagery vividness and behavioral or neural measures.

Therefore, the aim of the present study was to translate, culturally adapt, and conduct a preliminary psychometric validation of the Russian version of the Vividness of Tactile Imagery Questionnaire (VTIQ-RUS). Specifically, we evaluated its internal consistency, inter-item coherence, and test–retest reliability over a two-week interval in a sample of healthy adults.

## Methods

### Participants

A total of 150 healthy volunteers (93 females; mean age ± s.d. = 29.48 ± 13.16 years) participated in the study. Participants were recruited through online advertisements and word of mouth. Inclusion criteria included: (i) age ≥16 years, (ii) fluency in Russian, and (iii) absence of self-reported neurological or psychiatric disorders. No formal exclusion criteria beyond these were applied. The sample included a wide age range and was predominantly female (62%). Given the exploratory nature of the study, no stratified analyses by age or gender were conducted; however, this represents a limitation for generalizability and should be addressed in future research. The experimental protocol was approved by the Ethics Committee of Shenzhen MSU-BIT University. All the participants (*N* = 150) freely agreed to answer a questionnaire and gave informed consent.

### Materials

The original VTIQ, developed in English by Nierhausva et al. (2023), consists of 16 items that describe different everyday somatosensory situations (*e.g*., holding a cup, walking barefoot on grass, or feeling a comb on the head). The items were used with a 5-point Likert scale of *How clear and detailed is your mental imagery* (‘*no imagery*’ = 1, ‘*vague and unclear*’ = 2, ‘*relatively clear and vivid*’ = 3, ‘*very vivid*’ = 4, ‘*very detailed and as vivid as a real stimulus*’ = 5), as suggested by the authors of the original item pool. This structure allows the questionnaire to assess the clarity and detail of tactile imagery across a range of common tactile experiences, capturing individual differences in the vividness of mental somatosensory representations. The total VTIQ-RUS score is calculated by summing responses across all 16 items, with higher scores indicating greater vividness of tactile imagery. No reverse-scored items are included. The resulting total score can be used as a continuous variable in statistical analyses for research purposes.

The questionnaire was translated into Russian language. Two PhD-level researchers with advanced English proficiency (C1–C2) independently translated the 16 items from English into Russian. The translators then collaboratively compared their versions and resolved discrepancies to produce a single consensus translation. To ensure accuracy and conceptual equivalence, the consensus version was back-translated into English by two independent translators who were unfamiliar with the original questionnaire. The back-translation was compared with the original English version, and no meaningful discrepancies were identified, indicating that the Russian version retained the content and meaning of the original items. Finally, face validity was evaluated by having participants comment on item clarity, cultural relevance, and acceptability, ensuring that the items were understandable and appropriate for the Russian-speaking population. The complete set of questions in English and in Russian is provided in Supplementary Tables 1, 2.

### Procedure

The study was conducted in a quiet experimental room, where participants were tested individually under standardized conditions. Upon arrival, participants received a brief explanation of the concept of mental imagery, with particular emphasis on tactile imagery as the mental representation of touch sensations in the absence of physical stimulation. They were informed that the purpose of the study was to translate and validate the Russian version of the VTIQ.

Participants completed the questionnaire in a digital format, administered individually by the experimenter. For each item, the experimenter read the statement aloud, and participants were asked to vividly imagine the described tactile sensation and rate the clarity and detail of their mental image using the provided 5-point Likert scale. For items 3 (‘*holding a cup of warm coffee*’) and 5 (‘*feeling the comb on your head while combing your hair*’), participants were additionally asked to verbalize their imagined sensations to ensure that they were correctly performing the tactile imagery task. Standardized instructions were provided to ensure consistency across participants and sessions.

Participants were encouraged to comment on the clarity, cultural appropriateness, and comprehensibility of each item. Face validity was assessed qualitatively by collecting this feedback during questionnaire administration, with participants invited to report any ambiguities or difficulties in understanding the items. These remarks were used to identify potential issues in wording and to ensure that the translated items were interpretable and appropriate for the target population. No substantial problems were reported, and only minor linguistic adjustments were required, supporting the face validity of the instrument. It was explicitly stated that there were no right or wrong answers, and participants were encouraged to respond based on their subjective experience. All responses were collected anonymously, and participants were assured that their data would remain confidential and used solely for research purposes.

For assessment of temporal stability, all participants completed the questionnaire again after an interval of approximately 2 weeks under comparable testing conditions. Quantitative data from both administrations were used for psychometric analyses, including internal consistency, inter-item correlations, and test–retest reliability.

### Statistical analyses

All statistical analyses were conducted using custom Python scripts. The psychometric evaluation of the Russian version of the VTIQ included assessment of internal consistency, test–retest reliability, inter-item correlations, and factor structure.

Internal consistency of the 16-item scale was assessed using Cronbach’s *α* calculated separately for the first (t1) and second (t2) administrations using Pingouin library (Vallat, 2018). Alpha values ≥0.70 were considered acceptable, ≥0.80 good, and ≥0.90 excellent (Bland and Altman, 1997). Normality of the total scores was evaluated using the Shapiro–Wilk test. Since the distributions significantly deviated from normality, non-parametric methods were applied for test–retest comparisons. Test–retest reliability over a two-week interval was assessed using Spearman’s rank correlation between total scores at t1 and t2. Additionally, Wilcoxon signed-rank tests were used to examine potential systematic differences between t1 and t2 scores. Inter-item correlations were computed using Spearman correlation between all pairs of items. Factor analysis was conducted to examine the underlying structure of the VTIQ-RUS. Factorability of the scale was assessed using the Kaiser-Meyer-Olkin (KMO) measure of sampling adequacy and Bartlett’s test of sphericity. A KMO value ≥0.70 and a significant Bartlett test (*p* < 0.05) were considered indicative of suitability for factor analysis (Bartlett, 1954; Kaiser, 1974; Lloret et al., 2017). Exploratory factor analysis (EFA) was conducted to investigate the underlying structure of the scale and estimate factor loadings for each item. A one-factor solution without rotation was specified, and all analyses were performed using the FactorAnalyzer library in Python. Factor loadings ≥0.60 were interpreted as acceptable (Guadagnoli and Velicer, 1988), indicating meaningful contribution of each item to the latent construct of tactile imagery vividness. Construct validity was not assessed in the present study, as no additional measures were collected. This should be addressed in future research.

## Results

**Figure 1:**
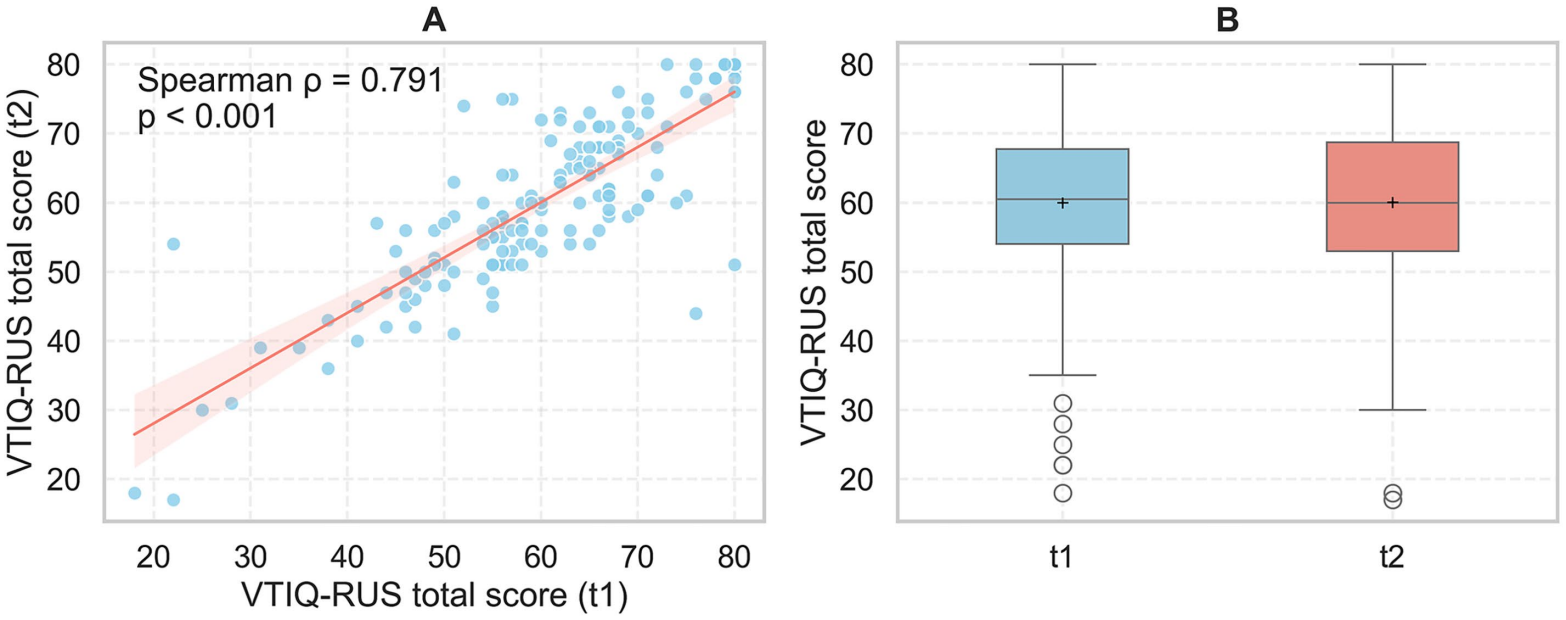
Test–retest reliability and score distribution of the VTIQ-RUS across first and second administrations. **(A)** Relationship between total VTIQ-RUS scores obtained during the first (t1) and second (t2) administrations. Each dot represents one participant; the solid line indicates the linear regression fit, and the shaded area represents the 95% confidence interval. Spearman’s *ρ* = 0.791, *p* < 0.001. **(B)** Boxplots summarizing total VTIQ-RUS scores at t1 and t2. Boxes represent the interquartile range (IQR), the horizontal line indicates the median, the “+” symbol denotes the mean, and whiskers extend to 1.5 × IQR from the quartiles.

The mean (*N* = 150) total VTIQ-RUS scores at the first administration (t1) were 59.97 ± 12.71 and at the second administration (t2) were 60.01 ± 12.33, indicating minimal variation between the measurements. Internal consistency of the scale was high, with Cronbach’s *α* values of 0.934 at t1 and 0.935 at t2. Normality tests indicated that total scores significantly deviated from a normal distribution (Shapiro–Wilk: W*t1* = 0.955, *p* = 0.0001; W*t2* = 0.967, *p* = 0.0011). Test–retest reliability was assessed using Spearman’s rank correlation, which showed a strong positive association between t1 and t2 total scores (*ρ* = 0.791, *p* < 0.001). The Wilcoxon signed-rank test revealed no significant difference between t1 and t2 scores (*W* = 4015.5, *p* = 0.88), with an effect size of *r* = 0.19 (Figure 1).

**Figure 2:**
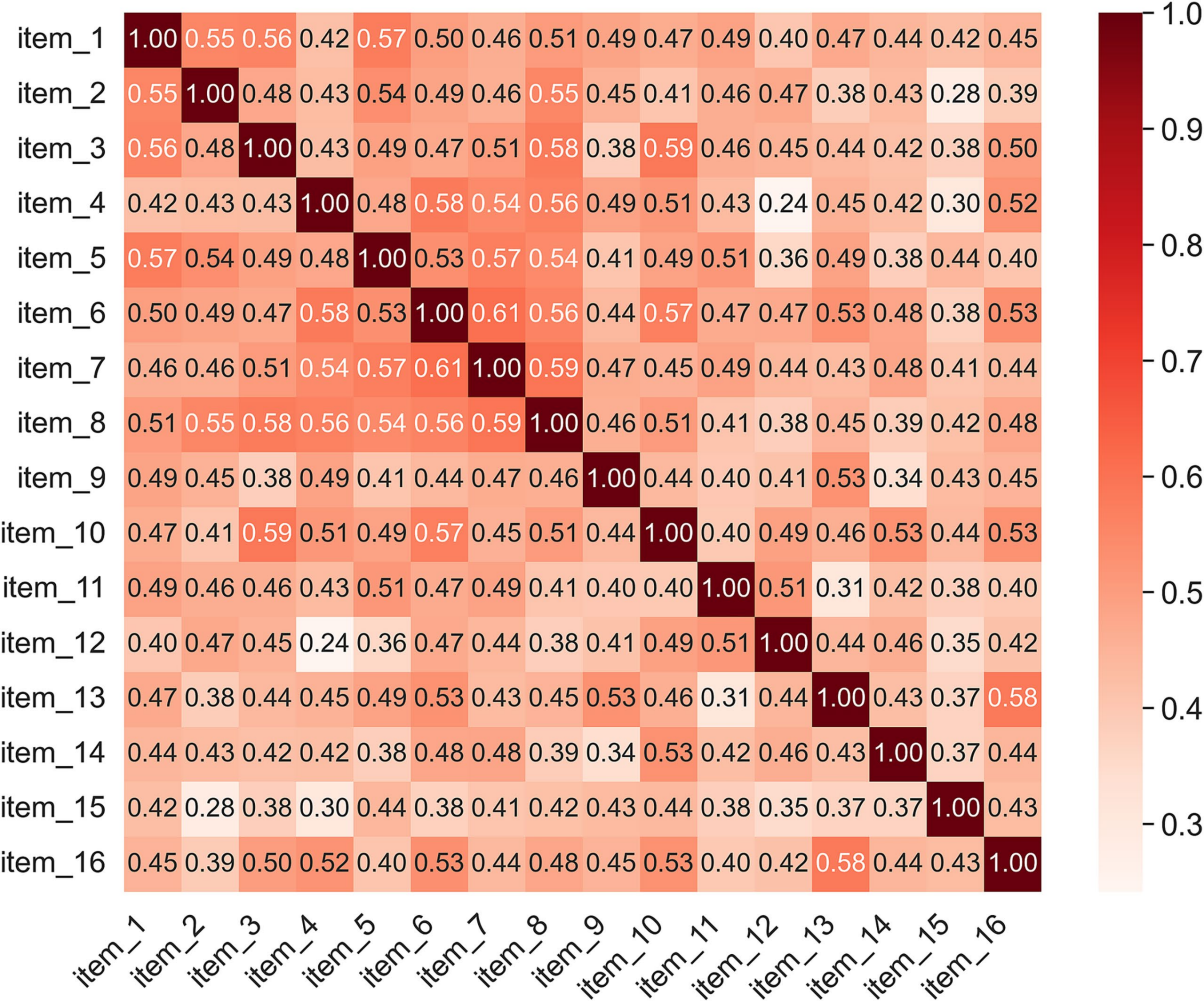
Spearman inter-item correlation matrix for the 16 items of the scale. Warmer colors indicate stronger positive correlations.

**Figure 3:**
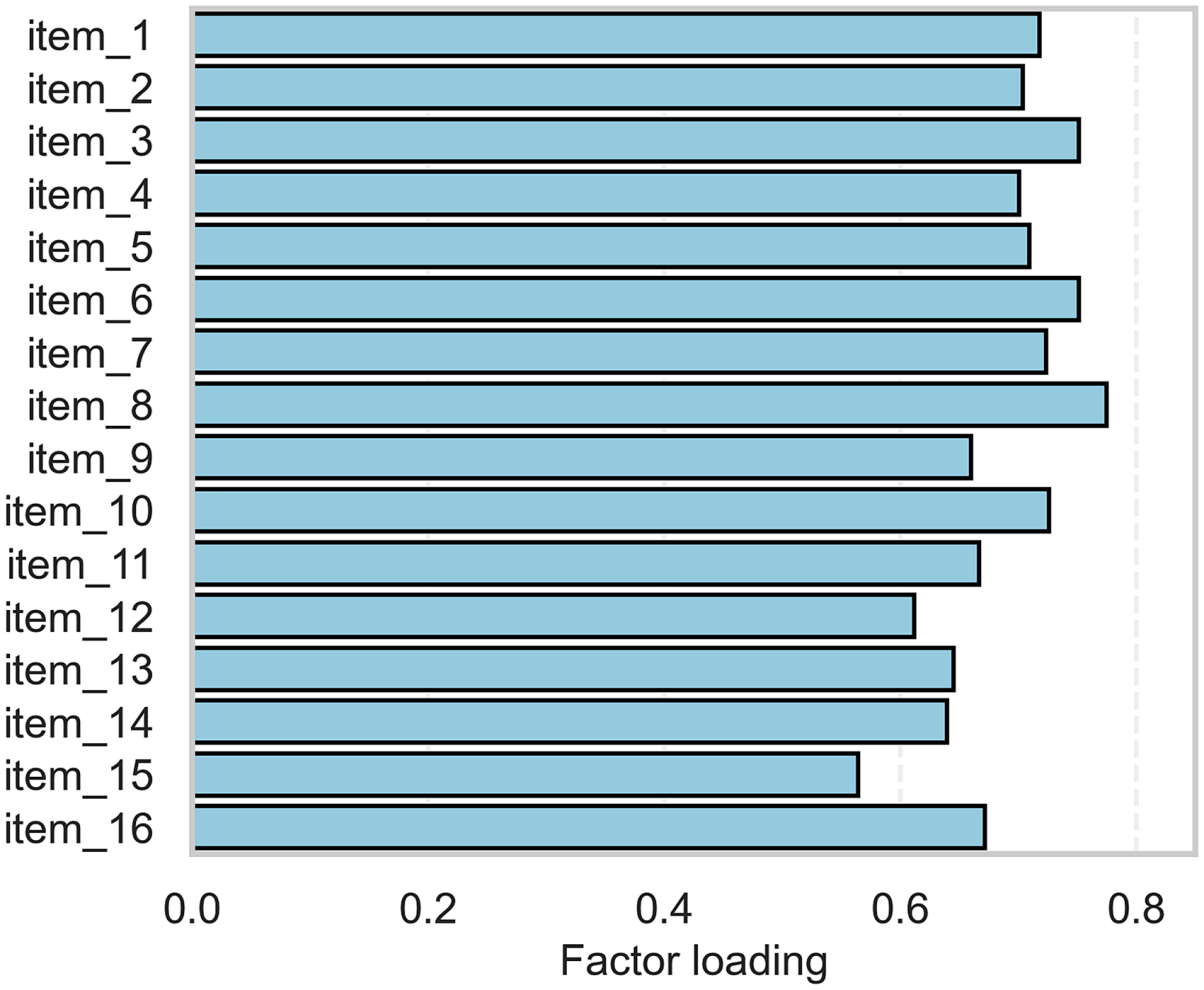
Standardized factor loadings for each item derived from the one-factor exploratory factor analysis (EFA) solution.

Inter-item correlations at t1 ranged from 0.241 to 0.608, with a mean of 0.459, suggesting moderate coherence among the items (Figure 2). Factorability of the scale was supported by a high KMO measure of sampling adequacy (KMO = 0.928) and a significant Bartlett’s test of sphericity (*p* < 0.001). A one-factor solution was specified based on the theoretical assumption that tactile imagery vividness represents a single latent construct, consistent with the structure of analogous mental imagery questionnaires (Marks, 1973; Gissurarson, 1992). This approach was further supported by the observed pattern of results, as all items loaded moderately to strongly on a single factor (loadings = 0.564–0.774) in the exploratory factor analysis, indicating a coherent unidimensional structure (Figure 3).

## Discussion

The present study provides the first psychometric evaluation of the Russian version of the Vividness of Tactile Imagery Questionnaire (VTIQ-RUS). Our findings suggest that the VTIQ-RUS demonstrates good internal consistency and temporal stability for assessing individual differences in tactile imagery vividness.

Total scores at the first (t1) and second (t2) administrations were highly similar (t1: 59.97 ± 12.71; t2: 60.01 ± 12.33), indicating minimal variation between repeated measurements. Internal consistency of the scale was high at both administrations (Cronbach’s *α* = 0.934 at t1 and 0.935 at t2), confirming that the 16 items reliably capture the construct of tactile imagery vividness. Inter-item correlations at t1 were moderate (range = 0.241–0.608; mean = 0.459), suggesting that each item contributes meaningfully to the overall scale without excessive redundancy.

Test–retest reliability over the two-week interval was strong, with a Spearman correlation of *ρ* = 0.791 (*p* < 0.001). The Wilcoxon signed-rank test indicated no significant difference between t1 and t2 total scores (*W* = 4015.5, *p* = 0.88; effect size *r* = 0.19), supporting the temporal stability of the instrument.

Factorability of the scale was supported by a high Kaiser–Meyer–Olkin measure (KMO = 0.928) and a significant Bartlett’s test of sphericity (*p* < 0.001), indicating that the data were suitable for factor analysis. Exploratory factor analysis using a single-factor solution revealed that all items loaded moderately to strongly on the factor (loadings = from 0.564 to 0.774), providing preliminary evidence for a unidimensional structure. However, as confirmatory factor analysis was not conducted, this structure should be interpreted as preliminary and requiring further validation. These findings are consistent with the conceptualization of tactile imagery vividness as a single latent construct and demonstrate that the translation preserved the integrity of the original scale.

Previous research has shown that tactile imagery can modulate sensorimotor brain networks (Yakovlev et al., 2023, 2024, 2026; Morozova et al., 2024; Miroshnikov et al., 2025). However, evidence linking individual differences in tactile imagery vividness to neural outcomes remains limited. The VTIQ-RUS may provide a standardized tool for assessing individual differences in tactile imagery vividness, which may help facilitate more systematic investigation of variability in future neurophysiological research.

While the current findings provide initial support for the reliability and structural coherence of the VTIQ-RUS, several limitations should be acknowledged. The sample size (*N* = 150) is adequate for preliminary psychometric evaluation and exploratory factor analysis, but larger samples are needed for confirmatory factor analysis and more robust validation. Importantly, construct validity was not assessed in the present study and should be addressed in future research.

## Conclusion

The VTIQ-RUS demonstrates good internal consistency and temporal stability, providing initial evidence of its reliability as a measure of individual differences in tactile imagery vividness. The results of the exploratory factor analysis suggest a unidimensional structure; however, this finding should be considered preliminary in the absence of confirmatory factor analysis. To our knowledge, the VTIQ-RUS represents the first Russian-language instrument specifically designed to assess the vividness of tactile imagery. By offering a standardized assessment tool, it enables more systematic investigation of tactile imagery in both experimental and applied research settings. Future research should extend the present findings by conducting confirmatory factor analysis in larger and more diverse samples, as well as by establishing construct validity. Such work will help clarify the utility of the VTIQ-RUS for investigating somatosensory imagery and its potential relevance for applied and clinical contexts.

## Data Availability

The raw data supporting the conclusions of this article will be made available by the authors, without undue reservation.
