# Supplementary material for: Preliminary psychometric validation of the Russian version of the vividness of tactile imagery questionnaire (VTIQ-RUS)
Source: Front Psychol. 2026 May 20;17:1814960. doi: 10.3389/fpsyg.2026.1814960 (PMC13230157; doi:10.3389/fpsyg.2026.1814960)
Supplement: Supplementary file 1 [file Supplementary_File_1.docx]

***SUPPLEMENTARY MATERIAL***

***Table 1.*** *Original english version of VTIQ*

| **Please think of the sensation of …** | **How clear and detailed is your mental imagery?** | | | | |
| --- | --- | --- | --- | --- | --- |
|  | No imagery (I only know that I am thinking about the sensation) | Vague and unclear | Relatively clear and vivid | Clear and vivid | Very detailed and as vivid as a real stimulus |
| 1. … your phone vibrating in your hand. |  |  |  |  |  |
| 2. … petting a cat. |  |  |  |  |  |
| 3. … holding a cup of warm coffee. |  |  |  |  |  |
| 4. … walking barefoot over pebbles. |  |  |  |  |  |
| 5. … feeling the comb on your head while combing your hair. |  |  |  |  |  |
| 6. … strolling barefoot on grass. |  |  |  |  |  |
| 7. … letting sand run through your fingers. |  |  |  |  |  |
| 8. … resting your forehead against a cool glass surface. |  |  |  |  |  |
| 9. … having a small stone in your shoe. |  |  |  |  |  |
| 10. … opening a plastic bottle cap by twisting it with your fingers. |  |  |  |  |  |
| 11. … brushing your teeth. |  |  |  |  |  |
| 12. … clapping your hands. |  |  |  |  |  |
| 13. … taking a cold shower. |  |  |  |  |  |
| 14. … filing your nails. |  |  |  |  |  |
| 15. … having your hair pulled by someone. |  |  |  |  |  |
| 16. … an ant crawling over your skin. |  |  |  |  |  |

***Table 2.*** *Translated russian version of VTIQ used in this study.*

| **Пожалуйста, подумайте об ощущениях …** | **Насколько ярки и детализированы ваши ментальные образы?** | | | | |
| --- | --- | --- | --- | --- | --- |
|  | Нет никаких образов (я знаю только то, что я думаю об ощущениях) | Расплывчатые и неясные | Относительно ясные и яркие | Ясные и яркие | Очень подробные и настолько же яркие, как и реальные ощущения |
| 1. …от телефона, вибрирующего в вашей руке. |  |  |  |  |  |
| 2. …когда вы гладите кошку. |  |  |  |  |  |
| 3. …когда вы держите чашку теплого кофе. |  |  |  |  |  |
| 4. …когда вы идёте босиком по гальке. |  |  |  |  |  |
| 5. …от расчёски на голове, когда вы расчесываете волосы. |  |  |  |  |  |
| 6. …когда вы гуляете босиком по траве. |  |  |  |  |  |
| 7. …когда сквозь пальцы сыплется песок. |  |  |  |  |  |
| 8. …от прикосновения лбом к холодному стеклу. |  |  |  |  |  |
| 9. …от маленького камешка в ботинке. |  |  |  |  |  |
| 10. …когда вы откручиваете крышку пластиковой бутылки. |  |  |  |  |  |
| 11. …когда вы чистите зубы. |  |  |  |  |  |
| 12. …когда вы хлопаете в ладоши. |  |  |  |  |  |
| 13. …когда вы принимаете холодный душ. |  |  |  |  |  |
| 14. …когда вы подпиливаете ногти. |  |  |  |  |  |
| 15. …когда кто-то потянул вас за волосы. |  |  |  |  |  |
| 16. …когда муравей ползет по вашей руке. |  |  |  |  |  |
